# Malunion of a Floating Knee: Overcoming the Challenges of Extra-articular Deformities With a Robotic-Arm-Assisted Total Knee Arthroplasty

**DOI:** 10.7759/cureus.68482

**Published:** 2024-09-02

**Authors:** Fathmath Iyaany Abdul Matheen, Juzaily F Leong, Rizal Abdul Rani, Muhammad Fathi Hayyun, Nor Hamdan Mohamad Yahaya

**Affiliations:** 1 Department of Orthopaedics and Traumatology, Universiti Kebangsaan Malaysia Medical Centre, Kuala Lumpur, MYS

**Keywords:** extra-articular deformity, robotic-assisted surgery, osteoarthritis, floating knee, total knee arthroplasty

## Abstract

Extra-articular deformities (EAD) can pose a challenge to surgeons during a total knee arthroplasty (TKA) surgery. Obtaining an acceptable post-operative hip-knee-ankle (HKA) angle may be difficult, especially in a limb with multiplanar deformities of both the femur and the tibia.

Our case is about a 66-year-old gentleman with a long-term deformity of his right lower limb secondary to malunion of the right femoral shaft and tibial shaft fractures. He initially presented with a right floating knee injury, 45 years ago, which was managed with conservative measures. He subsequently presented to us with ipsilateral knee osteoarthritis and underwent a robotic-assisted total knee arthroplasty surgery. Robotic- or computer-assisted total knee arthroplasty is an actively developing area and is gaining popularity among arthroplasty surgeons. In cases with severe extra-articular deformities such as in this case, robotic-assisted surgery can be superior to conventional surgery.

## Introduction

In patients with significant extra-articular deformities of the lower limb, doing a total knee replacement may prove to be a challenge. These deformities may be congenital, developmental, due to metabolic disorders, or even due to malunion of fractures [1]. When choosing to do a total knee arthroplasty in such patients, the predicament faced is the decision of whether to correct the deformity prior to the surgery, at the time of the surgery, or to leave the deformity uncorrected [2]. In the case of the latter, the plan for total knee arthroplasty should made in such a way as to compensate for the extra-articular deformities. 

In patients with extra-articular deformities of the lower limbs, the knee kinematics may be altered. The altered and asymmetrical forces acting on the knee joint may result in the deviation of the mechanical axis and early degenerative changes to the knee joint [3]. Proper preoperative templating is needed to decide the management of the extra-articular deformity. It may be especially useful to use robotic or computer assistance in the templating and the surgery. In a deformity that is distant from the knee joint, if the bone resections during templating can be made without interfering with the soft tissue structures, then an intra-articular compensatory correction can be chosen. This means there may be no need to directly address the deformities by doing a corrective osteotomy at the site of the deformity.

We will be discussing the case of a 66-year-old male with a right floating knee injury, with subsequent malunion of the fractures. A long-standing deformity of the femoral shaft and the ipsilateral tibial shaft, as in this case, can cause secondary osteoarthritic changes in the knee joint due to abnormal loading at the knee joint. This patient’s symptoms were addressed with a total knee arthroplasty surgery. In complex cases like this, the use of robotic or computer-assisted surgery could be beneficial to overcome some of the challenges.

## Case presentation

Our patient was a 66-year-old Malaysian man with underlying hypertension. He had a motor vehicle accident 45 years ago; he was a young man of 21 years of age at that time. He was riding his motorbike when a car coming from the front collided with his motorbike. After the incident, he was taken to another centre, and diagnosed with a closed fracture of the shaft of the right femur and an open fracture of the shaft of the right tibia. 

His condition was managed initially with a combination of wound care, skeletal traction, and a plaster of paris back slab. This was followed by an above-knee plaster of paris cast on the right lower limb for three months. After three months post-trauma, the cast was removed and he was allowed to ambulate. A total of eight months from the time of trauma, he completed his rehabilitation process and was able to return to work. Our patient then did not come for his follow-up appointments. Since then, even though he had some limitations of function such as not being able to squat down, he was still able to go to work and do his usual household work. 

He had been complaining of right knee pain for the past 10 years, and since then started using a single walking stick for ambulation. There had been progressive limitations in his day-to-day activities, including not being able to climb up and down the stairs, and not being able to drive for long distances. He was no longer able to participate in activities that he previously enjoyed, such as gardening. 

The pain progressively increased and he had to start using two walking sticks or a walking frame for ambulation for the past six months. The pain over the right knee increased in severity upon standing up, walking, and during activities that required turning. He did not have any night pain. He did not complain of any pain at the previous femur and tibia fracture sites. 

On examination, there was a varus deformity of the right knee joint and a 5-degree recurvatum of the knee joint as well as a significant recurvatum of the leg due to the tibia deformity. The knee joint flexion was limited to 105 degrees and posterior sagging of the joint was present. There was a scar over the shin from the initial laceration wound. There was also a scar over the posterolateral aspect of the knee, which according to the patient was a pressure wound due to the plaster of paris application during his initial treatment. 

**Figure 1:**
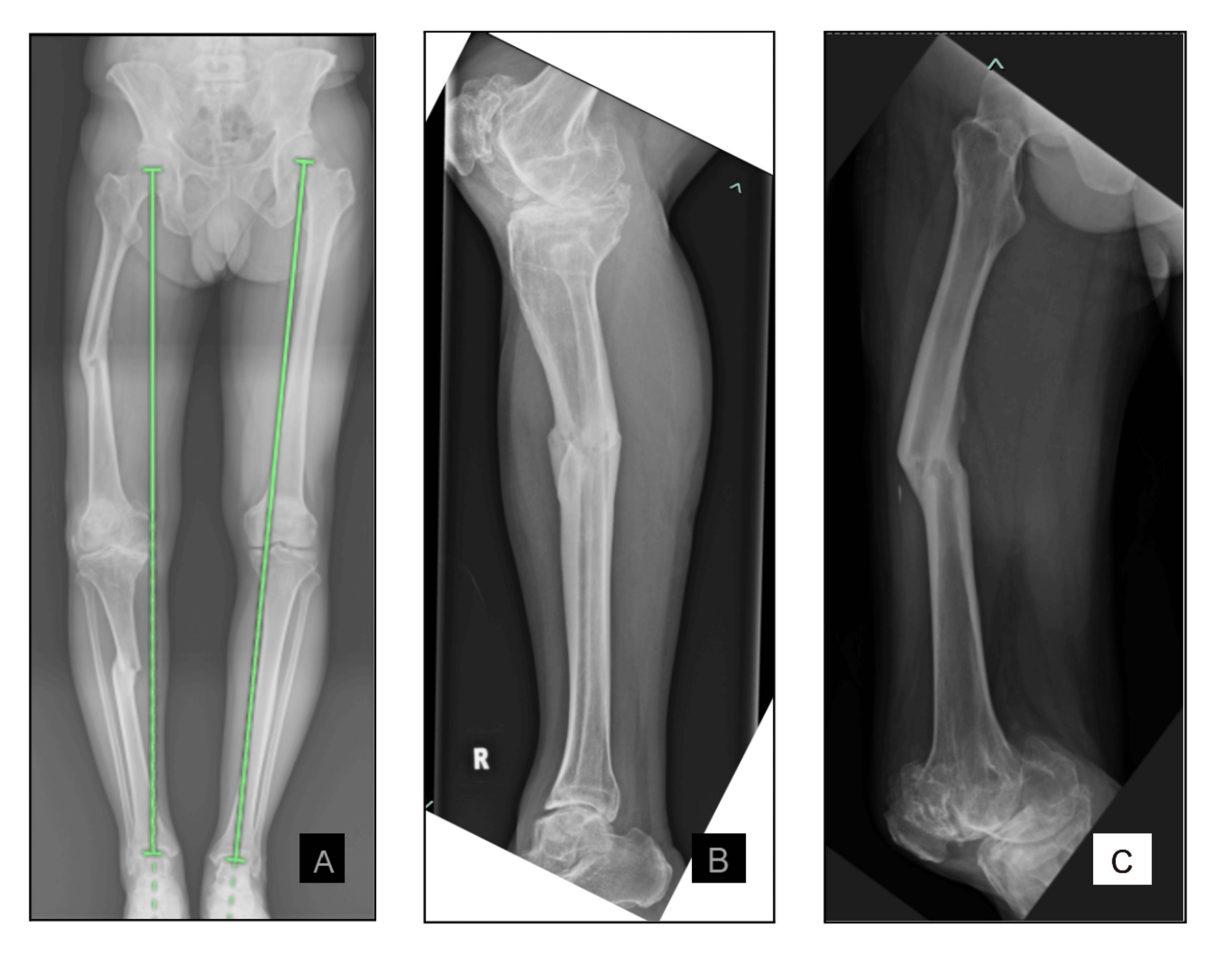
Preoperative radiographs. A) Bilateral lower limb scanogram of the anterior-posterior view, B) right tibia lateral view, and C) right femur lateral view Figure 1A shows the bilateral lower limb alignment during standing. This figure shows the coronal plane deformities of the femur and tibia shaft. The green lines indicate the mechanical axis of each lower limb. There is a deviation in the mechanical axis of the right lower limb. The right knee joint has severe osteoarthritic changes. Figure 1B is a lateral view of the right tibia showing the deformity of the tibia in the sagittal plane. Figure 1C shows the lateral view of the right femur showing the deformity of the femur in the sagittal plane. The lateral knee view of the right knee joint is also visualised with osteoarthritic changes.

Figure 1 shows the preoperative radiographs taken. It shows the deformities of the femur and the tibia. It demonstrates that the mechanical axis of the right lower limb is deviated medially and the right knee joint is in varus due to the deformities. It can also be appreciated from the preoperative radiographs that the joint space is greatly reduced with subchondral sclerosis and abundant osteophytes.

Surgical technique

Given the complexity of the case in view of the extra-articular deformities of the femur and the tibia, a robotic arm-assisted total knee arthroplasty was used. We used the Zimmer Biomet Robotic Surgical Assistant (ROSA) system (Zimmer Biomet Holdings, Inc., Warsaw, Indiana, United States). The robotic unit was set up in the operation theatre. The patient was then positioned supine, under general anaesthesia, and his right lower limb was cleaned and draped. A medial parapatellar approach was used, and a midline incision was given over the right knee and soft tissue dissected. 

The ROSA system was registered and the bone references and pins were placed. After acquiring the femoral and tibial landmarks, the initial knee condition was evaluated. This was achieved by moving the knee through a series of optically tracked movements. This evaluates and records the range of motion of the knee as well as detects any laxity of the knee.

The planning panel of the ROSA was then used to decide and set the necessary femur and tibia bone resection values and the implant component sizes. The robotic arm was then moved to the appropriate positions to help execute the planned resections accurately. Trialling was done, and intra-operatively, the knee was evaluated. 

For the femoral component, a size 8 standard posterior stabilising implant was used. A size E tibial tray was used with a 14 mm constrained posterior stabilised insert. After cementing of the femoral and tibial components and the tibial insert placement, final testing of stability and tracking was done. After thorough irrigation of the wound, the wound was closed in layers and an aseptic dressing applied.

**Figure 2:**
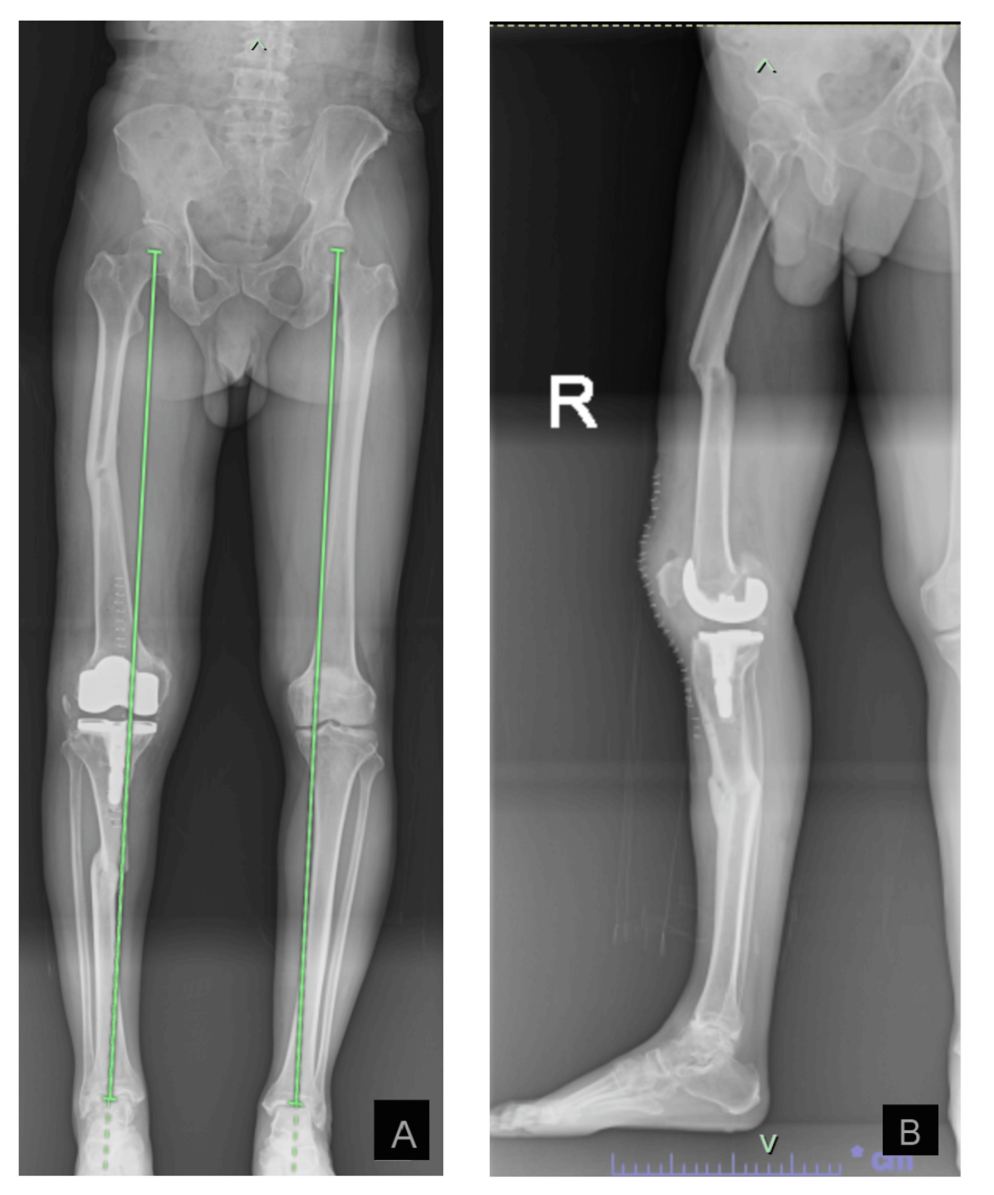
Post-operative radiographs. A) Anterior-posterior scanogram of the bilateral lower limbs and B) lateral view of the right lower limb Figure 2A is the anterior-posterior view of the bilateral lower limbs. The green line indicates the mechanical axis of the lower limb. The mechanical axis of the right lower limb is restored post-operatively. Figure 2B is the lateral view of the right lower limb. The sagittal alignment of the lower limb has improved post-operatively.

His immediate post-operative pain was well controlled. After six hours postoperatively, he was able to flex and extend his knee actively and was able to start ambulating with a walking frame. The deformity of his right lower limb had improved and the patient was quite satisfied. The post-operative check radiograph as shown in Figure 2 was acceptable and the mechanical axis of the right lower limb had been restored.

## Discussion

In the literature, there are several studies about the role of robotic-assisted surgery in dealing with extra-articular deformities of either the femur or tibia. A retrospective analysis was conducted in 2021 which included six patients who had coronal plane extra-articular deformities of the tibia ranging from 5 to 11 degrees [2]. They all underwent total knee arthroplasties successfully with restoration of the mechanical axis in all six patients, with no need for correction of the tibial deformity. A computer navigation system was used for four of the patients, and preoperative planning software was used for the other two. 

An advantage of robotic- or computer-assisted surgery is that it does not require using an intramedullary alignment rod for the femur [3]. This is especially advantageous in patients with femur deformities. Ding et al. did a finite element analysis study to pinpoint the relationship between femur deformities and the contact area and stress on the various areas of the knee [4]. This study demonstrated that when the angulation of the femur was increased from the neutral position to 10 degrees of varus, this gradually increased the contact area, and mean stress and maximum stress of the medial tibial cartilage, medial tibial subchondral bone, and the medial meniscus. In the case of changing the femur angulation to 10 degrees valgus, the findings were the opposite, and the stress increased in the lateral compartment. 

Prior case reports have reported success in using robotic arm-assisted total knee arthroplasty in extra-articular deformities of the femur or tibia. A similar case was described by Cook-Richardson et al., in which they reported a patient with a malunited diaphyseal femur fracture who developed secondary osteoarthritis of the knee, which was initially treated with a high tibial osteotomy [5]. As the pain progressively worsened even after the high tibial osteotomy, a robotic arm-assisted total knee arthroplasty was done. Upon following up six months postoperatively, the radiographs demonstrated good mechanical axis alignment. Alturki et al. also reported a case of a robotic-assisted total knee arthroplasty, which was done successfully in a patient with a malunited femur shaft fracture [6].

Kim et al. did a systematic review and meta-analysis to investigate the clinical and radiological outcomes of total knee arthroplasties when computer-assisted surgery is used in patients with extra-articular deformities [7]. In this meta-analysis including 14 studies, it was found that the overall outcomes were favourable with using computer-assisted surgery for even complex extra-articular deformities.

A randomised controlled trial including 144 patients, of which half the patients underwent conventional total knee arthroplasty, and the other half underwent robotic-assisted total knee arthroplasty was conducted by Tian et al. [8]. The results of the study demonstrated that there was a significantly lower post-operative hip-knee-ankle (HKA) angle deviation in the group that underwent robotic-assisted surgery among the patients with severe pre-operative lower limb alignment deviation.

There are few case reports which demonstrate that a robotic-assisted knee arthroplasty has satisfactory results in extra-articular deformities of either the tibia or femur. However, there were no cases described in the available literature of traumatic knee osteoarthritis secondary to an ipsilateral malunited femur and tibia fracture managed with robotic-assisted total knee arthroplasty. 

## Conclusions

Upon following up six months postoperatively, the patient’s pain score had reduced significantly and he was able to ambulate independently without any aids. However, he still needed to use a single walking stick occasionally, for long-distance ambulation. His right lower limb deformity had improved, and he reported an overall noteworthy improvement in function. He was quite happy with the outcome of the surgery.

This patient had a good surgical outcome with the use of a robotic-assisted total knee arthroplasty despite significant preoperative deformity. The use of robotic surgery helped access the mechanical axis more easily without the use of fluoroscopy. It also prevented complications which can arise from having to use an intramedullary femoral alignment rod in patients with a femur deformity. Moreover, it reduced the total surgery procedure time and hence intra-operative bleeding.
